# Andreas Bartels: *Wissenschaft*. de Gruyter: Berlin, 2021, 255 pp., €24,95 (paperback), ISBN: 9783110648249

**DOI:** 10.1007/s10838-023-09639-4

**Published:** 2023-06-07

**Authors:** Niels Linnemann

**Affiliations:** Institute of Philosophy, University of Bremen, Enrique-Schmidt-Str. 7, 28359, Bremen, Germany

In his new book *Wissenschaft*, Andreas Bartels sets out to justify the superior epistemic authority of science^1^ vis-à-vis other forms of knowledge endeavours. The case Bartels makes is compelling, not least because he proceeds in a sober manner, with lots of care for well-selected real life examples from vast areas of science.

Given the general title of the book and its inclusion in a series entitled ‘Foundational topics of philosophy’, it is helpful to clarify from the outset that the book is not—at least not primarily—a dedicated textbook on the philosophy of science. It could be fruitfully used this way, but a limited construal of the book in such a manner would fail to appreciate its overall argument for the special standing of science. Nor is the book meant to reconfirm the importance of science in the aftermath of COVID-19, despite the temporal coincidence of its publication: if at all, on Bartels’ view, an *explication* of the immense power of the sciences would be in order, as the book “came into being in a time, which showed the value of science for continuance and flourishing of society to everyone” (epigraph at the beginning of the book; my translation).

I summarise and assess the book Chapter by Chapter; as the book is only available in (elegantly written) German, the summary will be more detailed than otherwise necessary. I end with some general remarks about its strengths and weaknesses, as well as on the recommended audience.

Chapter 1 walks the reader through historical and somewhat familiar conceptions of science (Bacon, Descartes, Kant, Carnap, Popper and Kuhn). What struck me as most valuable about the Chapter (apart from its obvious stage-setting role) is how Bartels identifies continuities between each of these major players, such as when fleshing out the anticipation of Popper’s call for continued testing by Bacon. A pedagogical highlight is arguably the section on Kant, whose a priori conception of certain scientific statements are nicely illustrated by a rational reconstruction of Kant’s argument on how the Newtonian axioms follow from more general a priori statements.

In Chapter 2, Bartels puts forward what he considers the distinguishing *feature* of science qua knowledge endeavour: scientific theories are rich nets of theoretical terms; they only connect to non-theoretical terms by extending them beyond regions of joint applicability. It is in this theoretical nature of science that we are supposed to find the key reason why scientific knowledge can be so fruitful: by interrelating theoretical terms, even huge gaps between different phenomena are bridged, and empirical information is “transported” from one side of the net to another; this allows for laying out dependencies—and thus explanations and predictions—otherwise not at all identifiable.

As shown by Bartels exemplarily through recourse to the standard model of cosmology, findings made on one side of the net (say, on the background temperature of the universe) result in restrictions on the other side of the net (say, on the curvature of spacetime); the way how these findings restrict one another then triggers novel explorations and explanations. (In the current example, as spacetime curvature in turn puts restrictions on the allowed energy-matter-content, the findings on temperature eventually led to the postulation of new forms of energy contributions, including dark energy, thus correcting the previously known energy-matter-content.) Consequently, scientific theories can be attributed a productive nature—they naturally carry the seeds for their own development within themselves—that mere summaries of what we know would definitely lack.

The way Bartels reveals the importance of theoretical terms is convincing because it does not come at the cost of moving away from practice and the role of the empirical. To the contrary: Bartels’ cool take at practice (as expanded on in the course of this review) quite undeniably shows how theoretical scientific knowledge is. And instead of giving this the negative, disappointing ring typically associated wifth ‘theory-ladenness’ (as, e.g., presented in the dismissal of the empiricist-foundationalist dream), theoreticity comes out as the decisive strength of science.

Upon developing his understanding of science as a centrally ‘theoretical’ endeavour, standard accounts of scientific theory, models, and confirmation are introduced in the Chapter. Subtler points about theorising are made through case studies—in addition to the explication of the role of theoretical terms via the case from the standard model of cosmology, two further examples are put forward: a model of birds is brought up to concretely demonstrate that complexity and heterogeneity of a modeling situation may only allow for imprecise statements but do not thereby necessarily impede effective intervention based on the model. Secondly, a theory of representation for literature and art is used to exemplarily show that theories in the humanities may just as well have to face the ‘tribunal of sense experiences’—albeit in a less direct and admittedly vaguer manner.

Generally, much of the insight to be gained from this book—and much of its argumentative thrust—derives from its excellent case studies. How science works so well, can succeed, etc. is often best demonstrated, as Bartels knows and shows, by exemplification, and not by hubristic moves of oversystematisation.

Chapter 3 is dedicated to two central *tasks* of the sciences—explanation and discovery. The central thesis of the section is that these tasks are inseparably linked: explanatory hypotheses drive discoveries, and discoveries motivate new explanations.

Naturally, the Chapter starts with a presentation of standard accounts of explanation (including the deductive-nomological, interventionist, and pragmatist accounts); the sober nature of Bartels’ approach mentioned before is well testified here by how he works out that mathematical explanations can be incorporated into the deductive-nomological model, or that the deductive-nomological model—despite its limitations—remains an important special case, which more advanced explanatory accounts need to reduce.

In the next step, the traditional neglect of issues in the context of discovery is rehearsed under (the familiar) reference to the Popperian tradition on which the philosopher of science is indifferent to the origin of hypotheses, models and theories. Not very surprisingly, Hanson’s work (1965) on discovery strategies (in the context of Kepler’s work) is used to showcase an alternative tradition of systematic engagement with discovery. Another core systematic aspect of discovery discussed is Schindler’s (2015) distinction between discoveries of phenomena prior to their theorising (‘that-what’) and discoveries of already theoretically described phenomena (‘what-that’). The here is nothing left! ‘that-what’ scenario is illustrated with scrutiny through the case of Alzheimer’s observation of strange (*eigenartige*) cases of dementia with onset in patients much younger than those affected by the already known forms of senile dementia. But more than that: the subtle issue of whether Alzheimer is really the discoverer of the disease named after him is used to demonstrate the role of explanatory terms in driving further discoveries and the interconnection between discovery and explanation.

Now, the Alzheimer case is an archetypical case of discovery; and so rightly there are other kinds of discoveries Bartels draws our attention to (without claiming exhaustiveness). For instance, discoveries also occur upon improving or replacing explanations in an already familiar model. (This widely undervalued observation is made concrete under recourse to a dynamic segregation model that gives arguably counter-intuitive insights into why groups segregate even though individuals do not want to.) Furthermore, a vivid example case about the discovery of complex numbers continues Bartels’ theme that mathematics, when it comes to explanation and discovery, is not that different from other sciences after all.

In Chapter 4, the reader is presented with the central demarcation problems for science: the demarcation of scientific knowledge from everyday knowledge as well as from ‘higher’ forms of knowledge (as metaphysics). While stressing a general (rather uncontroversial) continuity between everyday knowledge and science, Bartels finds—as pointed out before in Chapter 2—the decisive difference in the former’s wide usage of theoretical terms and mechanisms. (Notably, a predecessor to Bartels’ take is for instance that of Sellars, with the latter’s distinction between scientific and manifest image.)

In connection to Hoyningen-Huene’s (2013) thesis of science as more systematic than everyday knowledge, Bartels’ stress on theoretical entities as a feature of science can then do real work: the higher systematicity of science (in whatever respect, really) is simply revealed as a consequence of the substantial usage of theoretical terms and mechanisms. The ‘traditional’ demarcation problem—namely from pseudo-science—of course brings up Popper’s falsifiability criterion; in this context, Bartels discusses Popper’s partly exaggerated charges of non-scientificity (e.g., towards psychoanalysis). The question what kind of metaphysics is not a science or in fact a (pseudo-)science is outsourced into a Chapter of its own.

Chapter 5 concerns metaphysics, or rather the metaphysics of science—one of Bartels’ own core research areas. To the charges of pseudo-science, Bartels responds with a positive conception of metaphysics as a science-related field concerned with meta-scientific terms—basic categories of high generality such as causality, spacetime and laws of nature—that have typically been relevant across scientific theories and disciplines, both for explanation as well as discovery. The basic justification for metaphysics despite all criticism is seen in the fact that scientific understanding does not only happen at the ground-level but also at highly abstract levels.

His own discussions of the metaphysical topoi of spacetime (including the absolutist-relationist debate, the hole argument and super-substantivalism), laws of nature (best system account of laws, necessitarian theory, dispositionalism), and causality are then indeed well-informed by science (in particular by physics), successfully fleshing out Bartels’ science-based understanding of metaphysics (or at least the physics-part of it). In particular, due to my own sympathies for Bartels’ unificatory understanding of scientific metaphysics, it is a bit of a pity that terms that are not traditionally used in metaphysics, but arguably highly successful meta-scientific terms, such as ‘universality’ or ‘resilience’, are not discussed as potentially ‘metaphysical’.

An innovative discussion is that on possibility and necessity, which takes into account recent work of Bartels on the difference between epistemic and objective possibilities, as well as variants of objective possibilities—all nicely demonstrated (from the viewpoint of the philosopher of spacetime, at least) alongside energy-conditions, equivalent formulations of general relativity and the principle of equivalence.

In fact, unhappy with Bird’s and Fine’s conceptions of metaphysical necessity in the face of insights from gravitational theories, Bartels proposes an intermediate conception: think of the world as equipped with certain fundamentally equipped properties as known from physics (contra Fine 2002, there is no possibility of, say, schmass instead of mass) but do allow for different gravitational laws as in fact studied with theories of gravity/spacetime (contra Bird 2005), there is real possibility for the laws to differ in how they link these fundamental properties). Examples of (fallible) statements of metaphysical necessities on this understanding are then to be found, again when restricting to the context of spacetime theories, in the form of the equivalence principle or energy conditions—i.e., statements that, *given all what we know today*, are true and will not be violated in the future either.

We are getting towards the end of the book, and so Bartels moves on to the implications of his structural understanding of science: in the first half of Chapter 6 we learn how the traditional goals of science—objectivity and truth—can be upheld. The first point is that science has to be held accountable with respect to an ideal of objectivity but importantly not of certainty. Close to practice again, Bartels argues that science follows such an ideal of objectivity through recourse to methods of objectification from practice: we learn that one concrete method from economics and the social sciences more generally is that of randomised controlled trials, which run interventions on a test group but not on an associated control group, allowing for testing underlying assumptions important for general understanding as well as for understanding the effect of specific policies. Another method—that of invariance—is from physics: physical claims formulated relative to an observer system are for instance made more objective by providing translation rules of the observer system’s statements into other systems; physical theories can typically be brought into a form that is completely invariant under such transformations. An important lesson from Hacking (1983) is rehearsed here: invariance under linguistic, measurement and general circumstances is central in assessing a scientific result as objective.

But Bartels continues not only to defend the objectivity of science but also its special relationship to truth. His line here is that although unproblematic truth criteria are hard to come by, if not impossible to have, it is not the case that a correspondence notion for truth is meaningless (at least not, if we want to grant meaning to a correspondence notion of truth in the case of everyday statements—as we usually do).

The second part of Chapter 6 opens up to discussions about how the methodological and epistemological character of science bears on its role in public disputes, the ethics of science, and ethics in science (including a section on robot ethics). Bartels importantly registers attacks on the authority of science not only from extreme science skeptics (including intelligent design proponents and climate change deniers) but also from ‘science-critical’ sociologists. Again, the conception of science as a net of theory and model is brought to the forefront, allowing for rendering any such form of fast nihilation and relativisation of the objectivity claims of science naive. The section on ethics of science is a good read but is mainly an engagement with famous texts by Weber (1904/1988; 1917/1988). The section on ethics in science quickly discusses the general question of limits to basic research prior to getting to the two classic examples of embryonic research and genome editing.

The final Chapter concludes the book as an exercise in justifying the special role of science for *Welterklärung* and practical orientation. Again by example, warnings are made about declaring the shielding of certain areas from scientific analysis as well as about the hope that science can provide a unified world view. After various reflections on the interface between science and philosophy, the book finally ends with the question of whether we should now, after all, believe in science. Given all the work done by Bartels up to this point, the reader will likely find herself convinced that, indeed, “For answering theoretical questions […] up to solving practical social problems, there is no better orientation than that provided by scientific methods. In this, but only in this sense, one should believe in science” (p. 216).

What really makes the book methodologically special and an outstanding contribution to the literature overall are the wonderfully pieced-together case studies, carefully (and never artificially) employed to flesh out Bartels’ conception of science. The work Bartels has put into digging out examples even far away from his own home turf of physics and mathematics—drawing for instance on the life sciences but even, in one detailed instance, on literature theory—is not only impressive but sets out a role model for good argumentation in the philosophy of science. (Some examples will nevertheless only be properly appreciable with some background in physics.)

One might say that certain points are developed quite quickly, that proposals such as that for a third type of metaphysical necessity, or claims that the increased systematicity of science is due to the higher theoretical nature, need to be fleshed out further to manifest their full convincing power. In particular, whether Bartels’ account of theories indeed pays tribute to sciences other than the natural and social sciences from which most examples are taken, will probably have to be discussed further (despite his nice case for empirical tests to representational theories of art, or the showcased continuities between natural sciences and mathematics). On the other hand, one might just as well take it to be a necessity of a project such as this one that not all details can be spelled out, and that such instances are rather welcome outlines of avenues for future research. (I support this second understanding here.)

Bartels’ excellent book is highly recommended to anyone with an interest in the prime question of what science is and in what sense it is uncontested by or even superior to other knowledge endeavours. I would also inflict the book as a necessary read on whomever doubts the systematic relevance of scientific discovery or has so far built up her (epistemological) understanding of science without a systematic appreciation for how science develops and gets developed. Due to its self-contained form, it can (and should very much) be used from intermediate undergraduate classes onwards, even for students without much prior immersion in the philosophy of science.

## Footnotes

1 I will use the translation ‘science’ for ‘Wissenschaft’ in the following. This means, however, that science is supposed to include mathematics, literature theory, parts of theology at least, etc. in the following—in line with the scope of the German word ‘Wissenschaft’ (plus-minus quarrels of demarcation) but contra its common usage in English language.
